# Racial Disparity in Ductal Carcinoma in Situ: Risk-Predictive and Actionable Biomarkers for Early Intervention

**DOI:** 10.3390/cancers18111794

**Published:** 2026-05-31

**Authors:** Dana Franklin, Padmashree Rida, Nikita Jinna

**Affiliations:** 1 City of Hope Comprehensive Cancer Center, Duarte, CA 91010, USA; dfranklin@coh.org; 2 Rowland Hall, Salt Lake City, UT 84102, USA; padmashreerida@rowlandhall.org

**Keywords:** ductal carcinoma in situ, racial disparities, prognostic biomarkers, tumor microenvironment, early breast cancer

## Abstract

Ductal carcinoma in situ (DCIS) is a non-invasive precursor to invasive breast cancer. DCIS incidence continues to rise, yet its clinical management remains constrained by the absence of reliable biomarkers that can adequately distinguish indolent lesions from those with high invasive potential, to circumvent over- or under-treatment. Black women with DCIS are significantly more likely to progress to invasive breast cancer, are disproportionately diagnosed with high-grade, hormone receptor-negative lesions, and experience elevated risk of recurrence and mortality relative to White women with DCIS. These disparities persist despite comparable access to screening and treatment, suggesting underlying biological and tissue microenvironmental factors. This review synthesizes emerging evidence implicating early molecular and systemic changes that may be driving the disparity in DCIS progression. We highlight racial distinctions in interconnected pathways involving Wnt/β-catenin signaling, metabolic and nutritional dysregulation, immune microenvironment remodeling, and cellular tolerance of genomic instability. We further discuss how epigenetic alterations, obesity-associated inflammation, and immune dysregulation may arise during the pre-invasive stage that intersect with social and environmental exposures to influence racial differences in lesion fate. We spotlight candidate biomarkers disproportionately associated with aggressive disease in Black women—including KIFC1, a mediator of centrosome clustering and genomic instability tolerance, and ACKR1/DARC, a regulator of chemokine gradients and immune trafficking—as potential drivers of progression-permissive states. This review advances an integrated, equity-informed framework for DCIS progression that links early tumor evolution to coordinated alterations in genomic instability, immune regulation, metabolic signaling, and stress-adaptive pathways. Importantly, we propose that DCIS progression is governed not by isolated molecular alterations but by coordinated programs that enable survival under genomic and immunologic stress. Current clinical risk assays, which primarily capture tumor-intrinsic proliferation and hormone signaling, do not fully resolve these pathways and may therefore incompletely reflect biologically meaningful racial disparities. This synthesis underscores the need for pathway-level, microenvironment-informed, and population-representative approaches to DCIS risk stratification. Advancing such frameworks will be essential for identifying actionable biomarkers, refining early intervention strategies, and ultimately reducing racial disparities in breast cancer outcomes.

## Simple Summary

Ductal carcinoma in situ (DCIS) is a high-risk breast lesion with highly variable risk of progression to invasive cancer, rendering clinical management challenging. Black women with DCIS experience disproportionately higher risk of progression to invasive breast cancer and higher rates of aggressive disease, recurrence, and mortality, suggesting that current risk stratification approaches fail to capture important biological differences. This review highlights emerging evidence that DCIS progression is driven not only by tumor-intrinsic features but also by coordinated interactions across multiple biological pathways, including genomic instability, immune dysregulation, metabolic changes, and Wnt signaling. We emphasize that these processes may be shaped by both biological and social factors, contributing to observed racial disparities. Additionally, we discuss candidate biomarkers, such as KIFC1 and ACKR1, that may help identify high-risk lesions and inform more precise, individualized management strategies. Improving DCIS outcomes will require integrating molecular, microenvironmental, and population-informed approaches to better predict progression risk and reduce inequities in breast cancer care.

## 1. Introduction

Ductal carcinoma in situ (DCIS) accounts for approximately 20–25% of all detected breast cancer cases globally and is typically associated with a favorable clinical outcome [1]. DCIS is a non-invasive proliferation of malignant epithelial cells restricted to the mammary ducts and represents an early stage in breast tumorigenesis [2]. However, DCIS can breach the ductal basement membrane, invade surrounding tissue, and progress to invasive ductal carcinoma (IDC) [3]. Left untreated, an estimated 25–60% of DCIS lesions will progress to invasive disease often unpredictably and over variable time scales [4,5]. To mitigate this risk, DCIS is often managed with aggressive interventions, including surgery and cytotoxic radiotherapy, which may lead to overtreatment in patients with indolent disease [1]. Interestingly, a previous study found that the 20-year breast cancer-specific mortality for DCIS patients was 3.3% and this percentage was not reduced in patients who received lumpectomy, by the inclusion of adjuvant radiotherapy to reduce invasive recurrence [6]. Although adjuvant radiation therapy does not improve patient survival rates, it is often included in treatment strategies in addition to lumpectomy. Thus, DCIS treatment occupies a paradoxical position in breast oncology; clinicians need to balance the risk of over- and undertreatment, with the reality that DCIS remains a precursor lesion capable of progressing to life-threatening invasive disease. The widespread use of mammographic screening has increased DCIS detection, yet clinical management remains challenging due to the lack of reliable risk-stratifying biomarkers capable of distinguishing indolent from progressive lesions [7]. Current risk assessments rely heavily on morphological and histopathologic features such as nuclear grade, necrosis, and lesion size [8,9], but these approaches fail to comprehensively capture the underlying molecular heterogeneity and the evolutionary potential that underlie DCIS progression. Indeed, emerging genomic and clonal analyses suggest that DCIS and invasive breast cancer frequently share a common ancestral lineage [4]. These insights have catalyzed a paradigm shift toward biologically informed risk stratification, exemplified by ongoing active surveillance trials such as COMET and LORIS, which aim to identify patients with indolent disease who may safely avoid surgery [10,11]. Yet, the absence of robust predictive biomarkers remains a critical barrier to implementing such precision approaches in clinical practice. Consequently, some patients may undergo unnecessary treatment, while others with biologically aggressive lesions may not receive adequate intervention.

Within this context of biological uncertainty, racial disparities in DCIS outcomes represent an additional and unresolved challenge. Black women diagnosed with DCIS are more likely to progress to invasive breast cancer relative to White women, as show in population-based studies [12,13] and supported by molecular analyses [14]. In addition to this elevated risk of progression, Black women experience a poorer prognosis overall, including a higher likelihood of DCIS recurrence and the development of second breast tumors, both ipsilateral and contralateral [15,16]. Further, Black women exhibit a higher risk of death following DCIS diagnosis compared to their White counterparts [17]. This racial inequity in outcomes is paralleled by racial differences in DCIS biology, as Black women disproportionately present with more aggressive lesion features, including larger tumor size, higher nuclear grade, and high-risk molecular subtypes [14,18]. At the same time, race is not a discrete genetic or biological variable, but rather a proxy for the cumulative effects of genetic ancestry, conditions of life, and structural inequities that become biologically embodied across the life course [19]. Disentangling these interacting influences is therefore essential to understanding the mechanisms underlying racial disparities. These disparities may therefore be the result of a multifactorial interplay of biological, environmental, and structural influences rather than a single cause. Collectively, these findings underscore significant racial disparities in DCIS progression and outcomes, highlighting the need to better understand the biological and clinical drivers underlying this inequity.

On a biological level, DCIS lesions in Black women more often display markers of aggressive molecular subtypes-such as elevated proliferation, hormone-receptor negativity, and basal-like gene expression profiles that point to intrinsic variation in tumor biology and the surrounding microenvironment [14,18]. Racial differences in immune infiltration, inflammatory signaling, and stromal remodeling may further shape lesion behavior and disparities in progression [20]. Beyond tumor-intrinsic mechanisms, social and environmental determinants including unequal access to screening, diagnostic follow-up, and treatment can amplify biological vulnerability [21,22]. Chronic psychological stress and systemic inequities are also linked to alterations in inflammation, genomic stability, and DNA damage response pathways that may influence both DCIS onset and its transition to invasive disease in certain populations relative to others [21,22]. Together, these intersecting factors underscore the need to move beyond traditional histopathologic grading and toward integrated molecular and systems-level approaches that can uncover biomarkers predictive of progression in high-risk populations. Such strategies are essential not only for refining DCIS management but for addressing the persistent racial disparities that shape breast cancer outcomes.

Here, we advance an integrated, equity-informed framework for understanding DCIS progression that moves beyond tumor-intrinsic features to incorporate the interplay between molecular pathways, immune and metabolic context, and systemic exposures that converge to drive observed racial disparities in DCIS progression. We describe the disproportionate burden of aggressive DCIS among Black relative to other racial groups and review evidence identifying potential actionable biomarkers and pathways that could be driving these ethnic differences. We also discuss key molecular and environmental axes that may be intersecting to drive these disparities, including Wnt signaling, nutritional influences, and immune modulation, and highlight emerging biomarkers that link these pathways to DCIS progression. By situating DCIS within a broader systems biology and disparities context, this review aims to identify actionable targets for early intervention and to advance precision approaches to identifying and managing high-risk DCIS.

## 2. Racial Disparities in DCIS

Racial disparities in DCIS outcomes are consistently observed but remain poorly explained at a mechanistic level. Black women show a markedly increased risk for progression to IDC compared to other ethnic groups. Population-based analyses revealed that Black women are more likely to develop subsequent invasive breast cancer irrespective of initial tumor subtype, underscoring a persistent and subtype-independent risk [12]. These disparities in DCIS progression are accompanied by differences in tumor aggressiveness. Black women are more frequently diagnosed with high-grade lesions and ER^−^/PR^−^ subtypes, which are associated with elevated risk of recurrence and invasion. In a large population-based cohort, Black women exhibited a significantly higher risk of developing ER^−^/PR^−^ invasive breast cancer or triple-negative breast cancer compared to White women, even after controlling for demographic and clinical covariates [13]. Disparities in mortality further highlight the racially disparate burden in DCIS. Non-Hispanic Black women demonstrate higher rates of breast cancer–specific mortality following DCIS diagnosis, as evidenced by the National Surgical Adjuvant Breast and Bowel Project (NSABP) trial, despite receiving comparable locoregional and endocrine therapies [23]. Additionally, Black women face a significantly increased risk of DCIS recurrence and second breast tumor development, both ipsilateral and contralateral, compared to White women [15]. Notably, African American race has been identified as an independent risk factor for local DCIS recurrence [24]. Importantly, these disparities persist even when access to screening and standardized treatment is accounted for [23]. These patterns have often been interpreted as evidence of intrinsic biological differences; however, such interpretations risk oversimplification, particularly given that “self-identified race” is a socially construct that incompletely captures underlying genetic ancestry, environmental exposures, and lived experience. Discordance between self-identified race and biogeographic ancestry, along with underrepresentation of diverse populations in genomic datasets, limits the resolution at which true biological drivers can be identified. As a result, studies that include self-identified race as a biological variable may both obscure meaningful heterogeneity within racial groups and overstate differences between them.

Longitudinal monitoring of DCIS cohorts reveals racial disparities in long-term outcomes. Black women exhibit elevated hazard ratios for subsequent ER^−^/PR^−^ invasive breast cancer (HR = 1.86) and increased risk of ipsilateral invasive tumors (HR ≈ 1.46) compared with White women [13,15]. Even in randomized trial settings with equalized treatment, non-Hispanic Black women exhibit higher distant recurrence (HR = 5.03) and breast cancer mortality (HR = 3.83) than non-Hispanic White counterparts [23]. Further analyses show that Black women with DCIS display increased all-cause mortality, cardiovascular death, and breast-cancer-specific death [25]. Collectively, these findings indicate that racial disparities in DCIS outcomes cannot be entirely explained by access or treatment differences and suggest that critical determinants of progression may lie not only within tumor cells but also within the surrounding microenvironment.

Differences in clinicopathologic presentation have also been reported, although findings are more variable across studies. Large database analyses indicate that Black women with DCIS are more likely to present with larger lesions and multicentric disease [26]. However, in contrast to invasive breast cancer, estrogen receptor negativity is not consistently more prevalent in DCIS among Black women, with reported patterns varying by age and cohort [27]. Similarly, high-grade DCIS is not uniformly enriched in Black women and has in some studies been observed more frequently among White women, suggesting that traditional histopathologic features alone do not fully explain observed disparities in outcomes [28]. Collectively, these findings underscore the limitations of relying solely on morphology-based risk assessment.

Given the multifactorial nature of these disparities, it is imperative to move beyond focusing solely on standardizing treatment delivery. Research must aim to identify and characterize intrinsic molecular and tissue microenvironmental determinants of progression. Integrative approaches including biomarker discovery, genomic profiling, immune microenvironment characterization, and analyses of social-biologic interplay are needed to stratify risk more accurately. Such efforts can inform personalized prevention, surveillance, and intervention strategies to reduce the racially disproportionate burden of DCIS.

## 3. Racial Differences in DCIS Biology

Recent advances in molecular profiling have begun to directly address longstanding questions regarding whether DCIS biology differs across populations. A landmark multi-omic analysis provided the first comprehensive evaluation of DCIS biology stratified by self-reported race, integrating gene expression, copy-number alterations, receptor status, and tumor microenvironment features in a well-annotated cohort of Black and White women with long-term follow-up [14]. Together with emerging complementary studies, these data suggest that biological differences associated with population groups and span multiple levels of tumor regulation. Importantly, several tumor-intrinsic features, including genomic instability and proliferative signaling, are more frequently observed in tumors arising in Black women and may be engaged early in lesion development. Enrichment of these programs may increase evolutionary potential and lower threshold for invasive transition, providing a biologically plausible basis for disparities in DCIS progression that persist even with access to care is comparable.

While many prior insights into population-associated tumor biology are derived from studies of invasive breast cancer, the convergence of these findings with DCIS-specific multi-omic data suggest that disparities in disease trajectory may be established earlier than previously appreciated. At the same time, it is important to recognize that most studies rely on self-identified race, which incompletely captures underlying genetic ancestry and molecular heterogeneity. Where available, ancestry-informed genomic analyses offer improved resolution of these differences in DCIS biology reflect coordinated alterations across tumor-intrinsic and microenvironmental systems that shape lesion evolution from its earliest stages, rather than discrete or deterministic genetic differences.

### 3.1. Gene Expression and Signaling Pathways

Strand’s multi-omic analysis identified distinct gene expression programs associated with both DCIS diagnosis and subsequent ipsilateral breast events in Black versus White women. These differences included enrichment of pathways related to proliferation, cellular stress responses, and inflammatory signaling [14]. Tumors from Black women demonstrated increased expression of immune- and inflammation-associated genes, including interferon-stimulated genes such as STAT1 and IRF1, as well as cytokine-related signaling components such as IL6 and CXCL8, consistent with prior transcriptomic analyses demonstrating enhanced immune-related gene expression signatures in tumors from Black women [29,30].

Upregulation of transcriptional regulators such as MYC and cell cycle-associated genes including CCND1 further supports heightened proliferative signaling [30]. In addition, activation of inflammatory signaling pathways, including NF-κB and JAK/STAT, may contribute to a more pro-inflammatory and transcriptionally active tumor state. These transcriptional programs suggest that differences in key signaling networks relevant to tumor growth and immune interaction may be present early in DCIS development. This co-enrichment of the afore-mentioned pathways is biologically significant. Rather than representing independent processes, proliferation and inflammation appear tightly coupled [31,32,33], suggesting a tumor state in which rapid cell division occurs within a pro-inflammatory milieu. Such environments are known to promote DNA damage, oxidative stress, and selective pressure for resistant clones—features associated with progression competence rather than indolence [31,32,33]. Importantly, this transcriptional state mirrors patterns observed in aggressive invasive breast cancers disproportionately affecting Black women, indicating (i) that some these programs may be initiated early (during the in situ stage) rather than acquired later, and that (ii) progression likely reflects selection rather than wholesale reprograming [34,35,36].

### 3.2. Copy-Number Alterations and Genomic Instability

Race-associated differences were also observed in genomic architecture, including variation in copy-number alteration (CNA) burden and chromosomal instability (CIN). Tumors from Black women exhibit higher levels of genomic instability, including overall CNA burden and a higher prevalence of somatic mutations in key tumor suppressor genes. For example, a large genomic studies incorporating ancestry-informed analyses have reported a higher prevalence of TP53 mutation in tumors from Black women (40–50%) relative to White women (~30%), alongside greater genomic instability overall [37,38,39].

Recent sequencing analyses of DCIS cohorts with long-term follow-up, using self-reported race as a proxy for ancestry, further suggest that DCIS lesions arising in Black women are enriched for genomic alterations associated with aggressive tumor behavior and poor clinical outcomes [14]. These alterations include recurrent disruptions in pathways governing cell proliferation, cell-cycle progression, DNA damage response, and genomic stability maintenance, supporting the hypothesis that biologic divergence may emerge early during pre-invasive disease evolution. Such findings are particularly notable because they indicate that molecular features commonly associated with invasive progression may already be established at the DCIS stage in certain populations.

These differences are accompanied by recurrent copy-number gains in regions harboring oncogenes such as MYC (8q24) and ERBB2 (17q12), as well as losses in tumor suppressor loci including TP53 (17p13) and RB1 (13q14), consistent with prior genomic analyses [39]. The presence of these alterations at the DCIS stage suggests that genomic instability and clonal diversity may arise earlier in tumor evolution in certain populations, potentially facilitating progression toward invasive disease. Importantly, such findings are increasingly interpreted within an ancestry-informed framework, as genomic variation captured in large datasets reflects underlying genetic diversity rather than socially defined race alone.

This has important evolutionary implications: DCIS progression is increasingly understood not as a linear acquisition of mutations, but as a process of clonal selection acting on pre-existing diversity. Somatic genomic differences may act in parallel with epigenetic priming to shape DCIS progression risk. Elevated CIN expands the pool of genetic variants upon which selection can act, accelerating the emergence of invasion-capable clones. In this context, differences in genomic instability represent differences in evolutionary potential, providing a possible mechanistic basis for disparities in progression risk and clinical outcomes.

### 3.3. Hormone Receptor and HER2 Signaling Differences

Differences in estrogen receptor (ER) and HER2 signaling were also evident. Black women are more likely to exhibit reduced expression of ESR1, consistent with the higher prevalence of ER-negative tumors. In the Carolina Breast Cancer Study, ER-negative disease was observed in approximately 39% of tumors in Black women compared to the 22% observed in White women, reflecting a substantially lower frequency of ER-positive tumors [40]. In parallel, ancestry-informed analyses, including those derived from The Cancer Genome Atlas, have identified a higher frequency of HER2-enriched breast cancer (OR = 2.22, 95% CI: 1.10–4.47) compared to White women, consistent with a shift toward more aggressive intrinsic subtypes [38,40]. Variability in downstream ER signaling targets, including PGR and BCL2, may further influence hormone responsiveness. In addition, increased expression of proliferation markers such as MKI67 has been reported in more aggressive invasive breast cancer subtypes, particularly basal-like, HER2-enriched, and Luminal B tumors compared to Luminal A tumors [40]. These signaling differences may influence intrinsic subtype classification, progression risk, and therapeutic response, and while they are most clearly defined in invasive breast cancer, emerging evidence suggests that key molecular programs underlying subtype identity may begin to emerge in pre-invasive lesions and contribute to early tumor evolution. These differences are best interpreted within a framework that considers both genetic ancestry and environmental context, as subtype distribution may reflect the convergence of biological and systemic influences rather than categorical race alone.

### 3.4. Tumor Microenvironment and Immune Signaling

Race-associated differences in DCIS biology include alterations in transcriptional programs related to inflammation and immune signaling, although detailed characterization of immune cell composition within DCIS lesions remains limited. Molecular analyses of DCIS have reported that lesions from Black women are more likely to exhibit gene expression patterns associated with aggressive behavior and increased risk of progression to invasive disease, including enrichment of proliferative and inflammatory signaling pathways [13]. These findings suggest that immune-related differences may be present early [14,20], even at the in situ stage, but are primarily detected at the level of bulk transcriptional programs rather than discrete immune cell populations.

Importantly, while immune activation and inflammatory signaling are implicated in DCIS progression risk, most studies of DCIS lack the spatial, cellular, and cytokine-level resolution required to define specific immune cell subsets or functional immune states. As a result, many of the detailed immune microenvironment features associated with racial disparities—such as increased macrophage and T-cell infiltration or elevated pro-inflammatory cytokine expression—have been characterized predominantly in invasive breast cancers rather than in DCIS itself [21,29,41,42].

## 4. Racial Differences in Biomarkers That Drive Invasiveness

As previously mentioned, Black women diagnosed with DCIS are at increased risk of developing subsequent invasive breast cancer, particularly aggressive subtypes, compared with White women [13,15]. While the molecular mechanisms underlying this disparity remain incompletely understood, the enrichment of high-risk genomic and epigenetic features in tumors from Black women provides a plausible biological framework linking early lesions to invasive progression. Multiple studies demonstrate that tumors arising in Black women are enriched for biomarker profiles associated with genomic instability, high-risk molecular subtypes, and poor clinical outcomes, suggesting that biological differences may contribute to disparities in DCIS disease progression and survival as summarized in Table 1 [40,43,44]. Rather than representing isolated or siloed mechanisms, these features may be better conceptualized as converging axes of invasiveness that act in a coordinated and reinforcing manner. Together, they may establish a progression-permissive state, characterized by enhanced proliferation, epigenetic deregulation, and immune evasion, that lowers the threshold for invasive transition. Experimental systems that capture this state include DCIS organoid models, patient-derived xenografts, and in vivo progression or spatial profiling models, which enable interrogation of epithelial–stromal and immune interactions during early tumor evolution. However, there remains limited availability of racially diverse or ancestry-informed DCIS models, representing a key gap in assessing population-level differences in these processes [45,46,47]. Importantly, these biomarkers do not merely serve as indicators of aggressive biology; they help define a selective landscape in which cells capable of tolerating genomic instability, sustaining proliferative signaling, and evading immune constraint are preferentially expanded, thereby facilitating evolutionary trajectories toward invasion.

### 4.1. TP53 Alterations in DCIS Progression

Alterations in the tumor suppressor TP53 are strongly associated with breast cancer progression and the acquisition of invasive phenotypes, as loss of p53 function disrupts DNA damage response pathways, promotes genomic instability, and facilitates aggressive tumor behavior [48,49,50]. Beyond its canonical role in maintaining genomic integrity, increasing evidence demonstrates that p53 also functions as a key regulator of anti-tumor immunity, linking genomic instability to immune surveillance. Loss-of-function TP53 mutations can impair antigen processing and presentation pathways, reduce tumor cell recognition by cytotoxic T lymphocytes, and suppress innate immune signaling, thereby promoting immune evasion even in the context of heightened mutational burden [51,52,53,54].

Genomic instability and immune signaling are increasingly recognized as tightly coupled processes. Chromosomal instability arising from TP53 loss can activate cytosolic DNA-sensing pathways and inflammatory signaling while simultaneously promoting immune evasion, creating a permissive inflammatory environment that supports tumor survival [51,54]. TP53 loss of function has further been shown to drive inflammatory signaling that facilitates the survival and expansion of genomically unstable clones rather than enforcing immune-mediated elimination [51,55]. Consistent with these mechanistic links, TP53 mutations can arise early during breast tumorigenesis and are detectable in a subset of pure DCIS lesions, with identical TP53 mutations frequently shared between in situ and matched invasive components. Full sequencing studies identifying identical TP53 mutations in DCIS and invasive carcinoma support a model of clonal evolution from in situ to invasive disease [56].

Against this backdrop of TP53-mutation-associated DCIS progression, comparative genomic analyses have reported a higher frequency of TP53 mutations and aberrant p53 signaling in tumors from Black women compared with White women, findings that have been most extensively characterized in invasive disease but likely reflect earlier divergence during pre-invasive stages [37,38]. Aberrant p53 expression is strongly associated with genomic instability, aggressive clinicopathologic features, and poor clinical outcomes, supporting its value as a marker of high-risk disease biology [48,50].

However, the extent to which TP53 alterations act as independent drivers of DCISprogression, rather than reflecting enrichment within aggressive molecular suptypes, remains incompletely defined. While mechanistic studies support a functional role for p53 loss in promoting genomic instability, immune evasion, and clonal expansion, direct evidence establishing TP53 alterations as causal determinants of progression in DCIS is limited.

Accordingly, TP53 dysregulation may be best conceptualized as both a marker of aggressive diseases states and a biologically plausible contributor to progression, with its precise role likely dependent on broader genomic and microenvironmental context. These underscore the need for DCIS-specific, longitudinal, and ancestry-informed studies to determine whether TP53 alterations independently predict progression risk (after adjustment for grade, receptor status, treatment, and biogeographic ancestry) or primarily reflect high-risk tumor biology.

### 4.2. Basal-like Phenotypes in DCIS Progression

Basal-like molecular phenotypes are associated with aggressive tumor behavior and a heightened propensity for rapid progression and invasion. These tumors are characterized by expression of basal cytokeratin’s such as cytokeratin 5/6 (CK5/6) and epidermal growth factor receptor (EGFR), along with loss of estrogen and progesterone receptor expression (ER^−^/PR^−^) [57,58,59]. Although less common than luminal subtypes, basal-like DCIS has been described as a high-grade in situ lesion that can serve as a direct precursor to invasive basal-like and triple-negative breast cancer, with frequent retention of basal marker expression across the in situ–invasive transition [60,61,62]. It therefore appears that lineage identity—and its associated aggressive phenotype—is established prior to invasion rather than acquired during progression. EGFR expression in DCIS has further been linked to proliferative signaling and molecular concordance with associated invasive tumors, supporting its role in early tumor evolution [4,63].

Population-based studies consistently demonstrate that Black women are disproportionately affected by basal-like and triple-negative breast cancer subtypes compared with White women, even after accounting for socioeconomic status, tumor stage, and clinical factors [64,65]. To the extent that basal-like molecular programs are operative in DCIS, enrichment of these phenotypes among Black women may contribute to an increased risk of rapid progression to invasive carcinoma, potentially through enhanced proliferative capacity, EGFR-mediated signaling, and lack of responsiveness to hormone-based prevention strategies [4].

### 4.3. Proliferative Drivers of DCIS Progression

Heightened proliferative signaling is a key driver of DCIS progression to invasive breast cancer, regulating cell cycle control and tumor growth [4,66]. Increased expression of cell-cycle-associated markers such as Ki-67 and dysregulation of the RB–CDK4/6 pathway—has been consistently associated with DCIS recurrence and progression to invasive disease [4,67]. Among these markers, elevated expression of the proliferation index Ki-67 has been one of the most reproducibly linked to increased risk of DCIS recurrence and invasive transition [68]. Notably, multiple studies have demonstrated higher Ki-67 expression in breast tumors from Black women compared with White women, suggesting greater proliferative capacity that may contribute to more aggressive disease behavior and earlier progression.

### 4.4. Epigenetic Drivers of DCIS Progression

In parallel, emerging evidence indicates that broader epigenetic landscapes differ by race in breast cancer, with most consistent differences observed at the level of DNA methylation-mediated gene regulation [69]. Genome-wide DNA methylation and transcriptomic analyses have identified race-associated epigenetic differences in promoter and CpG islands methylation affecting genes involved in cell-cycle regulation, differentiation, and tumor suppression, including key regulators of growth factor signaling such as LRIG1 and WWOX, with corresponding changes in gene expression [69,70,71]. Notably, population-specific differences in promotor hypermethylation at critical tumor suppressor loci have been well documented. Among younger women with hormone receptor-negative breast cancer, Black women exhibit significantly higher frequencies of promoter hypermethylation in multiple tumor suppressor genes, including HIN-1(SCGB3A1), TWIST, CCND2, and RASSF1A, with markedly higher rates of concurrent methylation across these loci, indicative of coordinated epigenetic silencing [72].

Functionally, these genes converge on key tumor suppressive and developmental pathways and help stabilize transcriptional programs that fuel aggressive phenotypes. RASSF1A is a well-established tumor suppressor that regulates cell cycle control, apoptosis, and microtubule stability with its epigenetic silencing is among the most frequent events in breast carcinogenesis [73,74,75]. HIN-1 encodes a secreted protein that functions as an autocrine inhibitor of epithelial cell proliferation and invasion and is frequently silenced through promotor hypermethylation in early-stage and invasive breast legions [76,77]. Similarly, methylation-associated loss of CCND2, a key regulator of the G1/S cell cycle transition, disrupts cell cycle control and promotes unchecked proliferation [78,79]. In contrast, TWIST, a central transcription factor regulating epithelial-to-mesenchymal transition (EMT), contributes to cellular plasticity, invasion, and metastatic progression in breast cancer [80,81]. Collectively, dysregulation of these genes reflects coordinated disruption of pathways governing cell cycle progression, lineage differentiation, and invasive phenotypes.

Importantly, emerging data suggest that such epigenetic alterations may arise prior to malignant transformation, with methylation changes detectable in benign breast lesions and associated with increased risk of progression, supporting a model in which methylation-mediated transcriptional silencing stabilizes proliferative, stem-like, or hormone-insensitive cellular states early in disease evolution [72]. Consistent with this, epigenetic differences are observed not only in invasive tumors but also in normal-adjacent and histologically normal breast tissue, supporting a race-associated field effect that may influence disease trajectory [22,82]. Genome-wide analyses have demonstrated that histologically normal breast tissues from Black and White women exhibit hundreds of differentially methylated CpG sites enriched in genes involved in cell survival, development, and intercellular signaling [83], suggesting that baseline epigenetic states may preconfigure transcriptional programs prior to tumor initiation.

Within this framework, these patterns can be interpreted through the lens of “weathering,” wherein chronic exposure to social, environmental, and structural stressors drives cumulative biological change over the life course. Repeated exposure, such as psychosocial stress, environmental toxicants, and structural inequities, can promote sustained epigenetic reprogramming through mechanisms including chronic inflammation, oxidative stress, glucocorticoid signaling, and altered DNA methyltransferase activity [84,85,86,87], with emerging evidence demonstrating that neighborhood-level structural factors such as redlining are associated with distinct DNA methylation patterns in breast tumors, thereby reinforcing the link between social context and tumor epigenomics [88]. These processes may reinforce early methylation-mediated silencing of tumor suppressor pathways, thereby accelerating progression from pre-invasive lesions to invasive disease. Accordingly, these epigenetic differences should not be interpreted as reflecting intrinsic racial biology, but rather as the biological embedding of lived experience, shaped by the interplay of genetic ancestry, environmental exposures, reproductive history, diet, and systemic inequities.

Epigenetic alterations may serve as a unifying mechanism linking environmental exposures, inflammation, and tumor intrinsic signaling by stabilizing transcriptional programs associated with sustained proliferation, immune evasion, and stem-like cellular states. Collectively, disparities in proliferative signaling and DNA methylation-driven epigenetic regulation, both at baseline and during tumor evolution, may represent coordinated biological mechanisms underlying the increased risk of DCIS progression and poorer outcomes observed among Black women.

## 5. Racial Disparity in Wnt Signaling and DCIS Progression

### 5.1. Mechanisms of Wnt-Driven Progression in Breast Cancer

Dysregulation of Wnt/β-catenin signaling has emerged as an important driver of breast cancer progression, invasiveness, and metastatic potential. In breast tumors, aberrant activation of Wnt pathways promotes proliferative signaling, disrupts epithelial organization, enhances cellular motility, and facilitates invasive behavior through induction of β-catenin-dependent transcriptional programs [89,90]. Consistent with these functional effects, activation of the Wnt/β-catenin pathway is particularly enriched in basal-like breast cancers and is strongly associated with adverse prognosis [91]. These data place Wnt signaling at the intersection of tumor aggressiveness and molecular subtype specification.

Unlike malignancies such as colorectal cancer—where activating mutations in APC or CTNNB1 are common—breast cancers rarely harbor genetic alterations in core Wnt pathway components. Instead, aberrant Wnt signaling typically arises through epigenetic and post-translational mechanisms. Foundational studies demonstrated that epigenetic silencing of secreted frizzled-related protein (SFRP) genes enables constitutive Wnt signaling in the absence of downstream mutations [90]. Subsequent work confirmed that epigenetic repression of multiple Wnt pathway regulators is prevalent in breast cancer, including SFRP family members and other negative regulators, establishing epigenetic derepression as a conserved mechanism of Wnt pathway activation in this disease [92].

Among these antagonists, SFRP1 is one of the most extensively characterized inhibitors of both canonical and non-canonical Wnt signaling. Promoter hypermethylation and transcriptional silencing of SFRP1, along with SFRP2, SFRP5, and DKK family members, have been consistently observed in breast cancer cell lines and primary tumors and are associated with enhanced β-catenin activity and aggressive tumor behavior [93]. Notably, aberrant methylation of SFRP1 has been linked to unfavorable clinical outcome, underscoring the prognostic relevance of Wnt antagonist loss in breast cancer progression [94]. Epigenetic deregulation of developmental regulators extends beyond direct Wnt antagonists, as promoter methylation-associated loss of *ID4* expression has been identified as a marker of breast cancer recurrence [95], highlighting a broader epigenetic landscape that favors tumor relapse and progression.

During progression from DCIS to invasive ductal carcinoma (IDC), accumulating evidence indicates that β-catenin localization shifts from a predominantly membranous pattern toward cytoplasmic and nuclear accumulation, reflecting activation of canonical Wnt transcriptional programs. Expression profiling of in vivo DCIS progression models identified B-cell lymphoma-9 (BCL9) as a critical molecular driver of invasion [96]. BCL9 functions as a co-activator of Wnt-stimulated β-catenin-mediated transcription, and its upregulation enhances nuclear β-catenin activity. Importantly, BCL9-driven Wnt activation may predispose DCIS lesions toward evolution into basal-like invasive breast cancers, positioning BCL9 as a potential biomarker of high-risk DCIS and a therapeutic target for invasion prevention.

**Figure 1:**
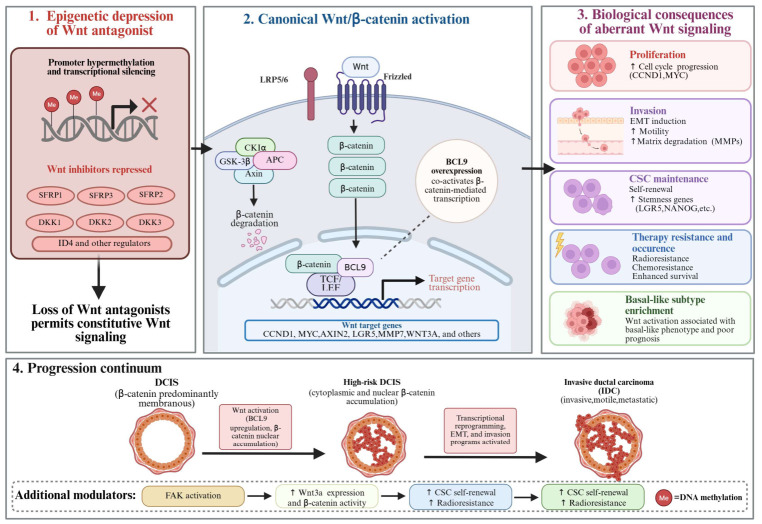
Mechanisms of Wnt-driven progression in breast cancer. This schematic illustrates how epigenetic dysregulation and canonical Wnt/β-catenin signaling drive breast cancer progression. Promoter hypermethylation of Wnt antagonists (SFRP1–3, DKK1–3, and ID4) leads to their transcriptional silencing, removing inhibitory control and enabling constitutive Wnt activation [89,91,92,93,94]. Wnt ligand binding to Frizzled and LRP5/6 disrupts the β-catenin destruction complex (AXIN, APC, GSK3β, and CK1α), resulting in β-catenin stabilization and nuclear accumulation. Nuclear β-catenin associates with TCF/LEF and co-activators such as BCL9 to induce transcription of target genes (CCND1, MYC, AXIN2, LGR5, MMP7, and WNT3A) that promote proliferative and stemness-associated programs [89,95,101]. Aberrant Wnt signaling drives key oncogenic phenotypes, including epithelial–mesenchymal transition (EMT), invasion, cancer stem cell (CSC) maintenance, therapy resistance, and enrichment of basal-like features associated with poor prognosis [90,96]. These processes contribute to progression from ductal carcinoma in situ (DCIS), characterized by predominantly membranous β-catenin, to high-risk DCIS with cytoplasmic and nuclear accumulation, and ultimately to invasive ductal carcinoma (IDC) with enhanced invasiveness and transcriptional reprogramming [95]. Additional modulators, such as focal adhesion kinase (FAK), further amplify Wnt signaling by increasing WNT3A expression, β-catenin activity, and CSC self-renewal while promoting radioresistance [97]. Arrows indicate directionality of pathway activation and disease progression. Me denotes DNA methylation.

Evidence further links Wnt signaling to cancer stem cell (CSC) maintenance and therapeutic resistance in DCIS. Canonical Wnt signaling is a key regulator of normal mammary stem cell self-renewal, and its dysregulation sustains CSC populations with enhanced invasive capacity and resistance to radiotherapy and systemic therapy [97]. In DCIS tissues, high levels of activated focal adhesion kinase (FAK) are associated with shorter time to recurrence [98]. Functional studies revealed a novel FAK–Wnt axis, whereby inhibition of FAK reduces Wnt3a expression, β-catenin levels, mammosphere formation, and self-renewal capacity while sensitizing DCIS cells to radiation. These data establish Wnt signaling as a central regulator of DCIS stemness and recurrence in pre-clinical and DCIS-relevant models [82]. Collectively, these data support a model in which early epigenetic derepression of Wnt signaling establishes a pro-invasive epithelial state while simultaneously promoting immune exclusion, thereby creating a permissive microenvironment for DCIS progression as summarized in Figure 1. We further posit that differential activation of Wnt signaling represents a mechanistically plausible contributor to racial disparities in DCIS progression.

### 5.2. Evidence for Early Wnt Dysregulation in DCIS

Although much of the mechanistic literature has focused on invasive breast cancer, emerging evidence suggests that Wnt pathway deregulation may occur early in breast tumorigenesis, including during the DCIS stage. Promoter hypermethylation of SFRP1 has been detected in DCIS lesions but is largely absent in normal breast epithelium, supporting the concept that loss of Wnt antagonism represents an early molecular event rather than a consequence of invasion [95]. Notably, SFRP1 methylation appears more frequent in higher-grade DCIS, which is associated with increased risk of progression to invasive disease [99,100].

**Figure 2:**
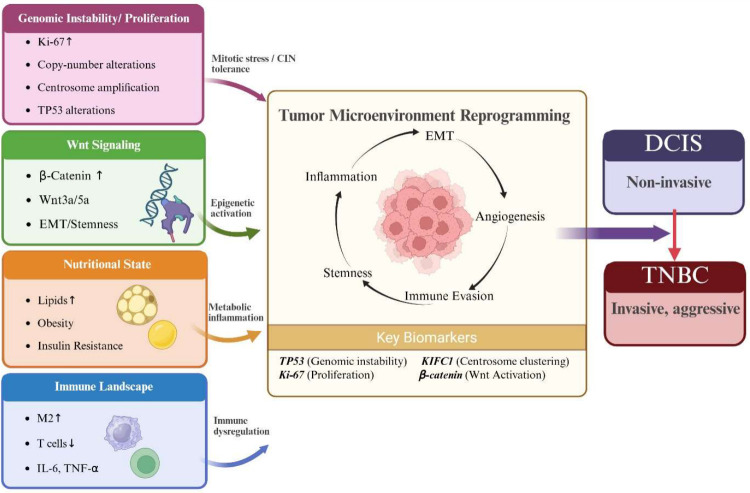
Conceptual model: interplay of Wnt signaling, metabolic state, and immune modulation in DCIS progression to TNBC. This conceptual model illustrates the coordinated interplay among tumor intrinsic genomic instability and proliferative stress, Wnt signaling, metabolic state, and immune modulation in driving progression from ductal carcinoma in situ (DCIS) to aggressive phenotypes such as triple negative breast cancer (TNBC). Early genomic alterations, including TP53 dysfunction, copy number alterations, centrosome amplification, and heightened proliferative signaling (Ki 67), generate intrinsic oncogenic and mitotic stress that predisposes epithelial cells to malignant evolution. In parallel, dysregulation of Wnt/β catenin signaling promotes epigenetic reprogramming, stemness, and epithelial–mesenchymal transition (EMT), while metabolic alterations—including obesity, lipid accumulation, and insulin resistance—contribute to a chronic pro inflammatory state. Concurrently, immune dysregulation characterized by reduced anti-tumor immune activity and increased pro tumorigenic signaling (e.g., M2 macrophage polarization and cytokines such as IL 6 and TNF α) further reshapes the tumor microenvironment (TME). These convergent pathways drive TME reprogramming marked by enhanced inflammation, immune evasion, angiogenesis, and cellular plasticity, creating conditions permissive for invasive progression. Key biomarkers associated with these processes include TP53 alterations, KIFC1 mediated centrosome clustering, Ki 67-associated proliferative signaling, and β catenin activation. Arrows indicate directionality of influence: solid arrows represent causal or functional contributions to tumor progression, while curved arrows within the TME denote reinforcing feedback loops among inflammatory signaling, immune evasion, and stemness. The gradient arrow from DCIS to TNBC reflects increasing disease aggressiveness. This model is informed by prior studies linking Wnt/β-catenin signaling, metabolic dysregulation, and immune microenvironment remodeling to breast cancer progression and aggressive phenotypes [85,86,87,102,103,104,105,106,107].

Despite these observations, direct evidence of downstream Wnt activation in DCIS remains limited. Few studies have systematically examined nuclear β-catenin localization or expression of canonical Wnt target genes in DCIS specimens, limiting definitive conclusions regarding the functional consequences of Wnt antagonist silencing at the pre-invasive stage. Nonetheless, existing data supports a model in which early epigenetic deregulation of Wnt pathway regulators may predispose DCIS lesions to invasive progression. Collectively, these pathways highlight the dynamic interplay between Wnt signaling, metabolic influences, and immune modulation in driving DCIS progression and tumor microenvironment reprogramming as depicted in Figure 2. Importantly, although these observations suggest that Wnt pathway dysregulation may occur in early in tumorigenesis, the extent to which such alterations differ across racial groups at the DCIS stage remains largely uncharacterized. As such, the proposed contribution of Wnt signaling to racial disparities in DCIS progression should be considered hypothesis-generating and requires validation in diverse, well-annotated cohorts.

### 5.3. Racial Disparities in Wnt Signaling

Although large scale studies directly examining Wnt pathway activation in DCIS stratified by race are lacking, evidence from invasive breast cancer and related biological context suggest the potential for race-associated differences in Wnt signaling activity. These observations provide a biologically plausible framework but remain indirect and should be therefore interpreted cautiously in the context of DCIS. In particular, studies in TNBC report enhanced Wnt/β-catenin pathway activation in tumors from Black women compared with White women, which has been proposed to contribute to more aggressive tumor phenotypes and poorer clinical outcomes [59,108]. Complementing these findings, genetic and transcriptomic analyses indicate that components of the Wnt signaling pathway and its regulatory networks may vary by ancestry, as demonstrated in studies of cancer susceptibility and pathway-level regulation across diverse populations [109].

Given the central role of epigenetic mechanisms in regulating Wnt pathway activation—and growing evidence that environmental, inflammatory, and psychosocial stress–related exposures can shape epigenetic landscapes—dysregulated Wnt signaling represents a biologically plausible contributor to racial disparities in breast cancer progression [95,102,110]. Addressing this important knowledge gap will require integrative studies in racially diverse DCIS cohorts that combine epigenetic and transcriptional profiling with longitudinal clinical outcome data to determine whether differential regulation of the Wnt pathway contributes to disparities in progression and invasiveness.

Future studies should prioritize integrative, ancestry-informed analyses combining epigenomic profiling, spatial transcriptomics, and immune phenotyping in DCIS cohorts with longitudinal follow-up. Such approaches will be essential to determine whether differential Wnt pathway activation represents an early, targetable driver of progression and whether it can serve as a biomarker for risk stratification across diverse populations.

### 5.4. Racial Disparity in Wnt-Induced Immune Modulation

Oncogenic signaling pathways that drive epithelial cell proliferation and fate determination also exert substantial influence over the tumor immune microenvironment. As a central regulator of mammary gland development and early breast tumorigenesis, Wnt signaling occupies a pivotal intersection between epithelial transformation and immune regulation. Across multiple cancer types, activation of canonical Wnt/β-catenin signaling has been associated with immune-excluded phenotypes, characterized by reduced dendritic cell recruitment, impaired antigen presentation, and diminished cytotoxic T-cell infiltration, thereby fostering immune evasion [103,104]. Although these mechanisms have been most extensively characterized in invasive cancers, emerging evidence suggests that Wnt-mediated immune modulation may be initiated at earlier, pre-invasive stages of disease.

A new paradigm for tumor immune escape was established by studies demonstrating that tumor-intrinsic activation of β-catenin signaling actively excludes antitumor immune cells from the tumor microenvironment. In this model, β-catenin-driven transcriptional programs suppress recruitment of key antigen-presenting cells, thereby preventing effective priming and infiltration of cytotoxic T cells and promoting immune escape [103]. Importantly, correlative analyses across human cancers have confirmed that Wnt/β-catenin pathway activation is strongly associated with immune exclusion, independent of tumor lineage, indicating that this mechanism represents a conserved feature of oncogenic signaling rather than a tumor-type–specific phenomenon [104].

Beyond immune exclusion, Wnt signaling has been implicated more broadly in shaping immunosuppressive tumor ecosystems. Comprehensive reviews have shown that aberrant Wnt activation can promote resistance to immune-mediated tumor control by fostering regulatory T-cell and myeloid-derived suppressor cell programs, altering chemokine gradients, and limiting responsiveness to both immunotherapy and conventional anticancer treatments [111]. Together, these findings highlight Wnt signaling as a central coordinator of epithelial transformation, therapeutic resistance, and immune evasion.

Although Wnt-driven immune modulation has been characterized most extensively in invasive malignancies, these mechanisms provide a biologically grounded framework for understanding how immune exclusion may arise earlier during tumor evolution. DCIS exhibits marked heterogeneity in immune composition, ranging from immune-enriched to immune-cold lesions, suggesting that oncogenic signaling pathways active during DCIS progression may influence immune engagement prior to invasion. Given the established roles of Wnt signaling in promoting epithelial plasticity, stemness, and invasive progression in breast cancer, sustained or dysregulated Wnt activation may simultaneously drive epithelial proliferation while constraining effective immune surveillance during early disease stages.

Importantly, racial disparities in Wnt pathway activation observed in invasive breast cancer provide a mechanistically plausible framework for understanding race-associated differences in immune regulation and disease progression. If Wnt/β-catenin signaling is more frequently activated, initiated earlier, or more persistently sustained in tumors arising in Black women, such differences could contribute to immune-excluded microenvironments even at the stage of DCIS. Collectively, these observations support a model in which enhanced Wnt signaling exerts coordinated effects on epithelial and immune compartments, promoting tumor progression while suppressing antitumor immunity. While direct evidence in DCIS remains limited, this framework offers a testable link between oncogenic signaling disparities, immune surveillance, and racial inequities in breast cancer outcomes.

## 6. Racial Disparity in Tumor Immune Microenvironment and DCIS Progression

### 6.1. Immune Dysregulation and DCIS Progression

DCIS is increasingly recognized as an immunologically active lesion, with immune cell infiltration evident at this pre-invasive stage of breast tumorigenesis. Histopathologic and transcriptomic analyses have demonstrated the presence of diverse immune populations within DCIS lesions, including T lymphocytes, B cells, macrophages, and neutrophils, with immune composition varying by lesion grade and molecular features [112,113]. High-grade DCIS, which is more likely to progress, is consistently associated with high levels of tumor-infiltrating lymphocytes (TILs), macrophage density, and inflammatory signaling relative to low-grade lesions and adjacent normal epithelium [112,114]. Importantly, immune infiltration in DCIS does not uniformly reflect effective anti-tumor immunity. Rather, emerging evidence suggests that the immune microenvironment may be functionally heterogeneous, encompassing both cytotoxic and immunosuppressive elements that influence lesion fate. Studies examining the transition from in situ to invasive disease indicate that immune escape mechanisms may be engaged early, including altered antigen presentation and immune cell polarization that favor tumor persistence [114]. These findings suggest that immune landscape at the DCIS stage may play a critical role in shaping progression risk, although longitudinal and mechanistic data remain limited.

Beyond tumor-intrinsic alterations, accumulating evidence highlights that differences in the immune microenvironment play a critical role in the transition from DCIS to invasive breast cancer. Comparative analyses demonstrate that DCIS lesions generally exhibit a more organized but variably active immune milieu, whereas invasive carcinomas are characterized by increased immune infiltration coupled with immunosuppressive remodeling, including enrichment of regulatory T cells, tumor-associated macrophages, and exhausted T-cell phenotypes. Notably, studies have shown that while immune cell density may increase with invasion, the functional orientation of these cells shifts toward tumor-promoting states [115]. In addition to immune cell composition, growing evidence implicates the myoepithelial–immune interface as a critical regulator of DCIS progression, as alterations in myoepithelial integrity, cytokine signaling, and antigen-presentation capacity can reshape immune cell recruitment and function, thereby facilitating immune escape prior to overt invasion [116,117].

Emerging biomarker-driven investigations further suggest that specific immune features within DCIS lesions may predict progression risk. For example, elevated stromal tumor-infiltrating lymphocytes (TILs), increased PD-L1 expression, and the presence of immunosuppressive myeloid populations have been associated with high-risk DCIS and subsequent invasive recurrence [118,119]. Additionally, transcriptional and spatial profiling studies have identified immune-related gene signatures linked to chronic inflammation, impaired antigen presentation, and immune evasion as key determinants of progression potential. These findings are supported by broader reviews and cohort analyses demonstrating that disruption of immune surveillance and establishment of a permissive tumor microenvironment are early and necessary events in DCIS progression [66,117,120,121]. These findings underscore the importance of immune contexture not only as a distinguishing feature between in situ and invasive disease, but also as a promising source of prognostic biomarkers and therapeutic targets within a broader immune–metabolic–oncogenic signaling framework as summarized in Table 2. Collectively, these findings support a mechanistic model in which early immune dysregulation in DCIS—characterized by concurrent inflammatory activation and immune suppression—creates a selective microenvironment that permits persistence and expansion of high-risk clones, thereby increasing the likelihood of invasive progression.

### 6.2. Racial Disparities in Immune Landscape and DCIS Progression

Emerging evidence suggests that immune contexture is an important determinant of DCIS progression and a potential contributor to racial disparities in breast cancer outcomes. Studies of DCIS progression have shown that the transition from in situ to invasive disease is associated with a shift from a variably active immune milieu toward immunosuppressive remodeling, characterized by enrichment of regulatory T cells, tumor-associated macrophages, T-cell exhaustion, and increased immune checkpoint signaling, including PD-L1 expression [115,118,119]. These immune features have been proposed as biomarkers of high-risk DCIS and may reflect early changes that predispose lesions to invasive progression.

Direct race-specific immune profiling within DCIS lesions remains limited. However, several immune features linked to DCIS progression risk overlap with immune phenotypes that have been shown to differ by race in invasive breast cancer. Tumors from Black women with invasive disease exhibit distinct immune landscapes, including altered B-cell composition, increased immunosuppressive myeloid and regulatory immune populations, and heightened inflammatory signaling [21,122]. These parallels suggest that race-associated differences in immune biology observed in invasive tumors may build upon earlier immune-related differences present at the in situ stage, even if they are not yet fully resolved at the cellular level in DCIS.

**Figure 3:**
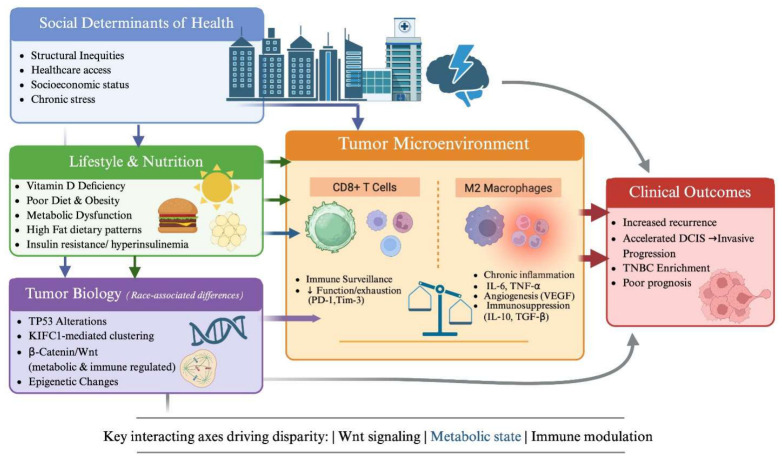
Conceptual framework linking multilevel determinants to racial disparities in DCIS progression and outcomes. This conceptual model illustrates how upstream social determinants of health—including structural inequities, healthcare access, socioeconomic status, and chronic stress—shape downstream lifestyle and nutritional exposures such as vitamin D deficiency, diet quality, obesity, and metabolic dysfunction, which in turn influence tumor biology and the tumor microenvironment. These exposures contribute to context-dependent tumor-intrinsic alterations, including TP53 mutations, KIFC1-mediated centrosome clustering, β-catenin/Wnt pathway activation, and epigenetic reprogramming, that favor tumor progression. Tumor-intrinsic and metabolic changes intersect within the tumor microenvironment (TME), shifting immune balance away from effective anti-tumor surveillance (e.g., CD8^+^ T cell activity) toward a pro-tumorigenic, inflammatory state characterized by M2 macrophage polarization and elevated cytokines such as IL-6 and TNF-α. Crosstalk among metabolic, immune, and Wnt signaling pathways reinforces immune dysregulation and tumor-promoting conditions, ultimately driving adverse clinical outcomes including increased recurrence risk, accelerated progression from DCIS to invasive disease, enrichment of aggressive subtypes such as triple-negative breast cancer (TNBC), and poorer prognosis; arrows indicate directionality and interaction across scales, with hierarchical influences flowing from upstream determinants to downstream biological effects, bidirectional crosstalk and feedback loops within the TME, and converging pathways reflecting the cumulative impact of these processes on disease progression. This framework is informed by prior studies on tumor microenvironment dynamics, immune dysregulation, metabolic signaling, and social determinants of health in breast cancer progression and disparities [102,103,104,105,106,107,125,126,127,128].

Mechanistically, variation in immune-related signaling pathways—such as chemokine-mediated immune cell recruitment and inflammatory cytokine signaling—may contribute to differences in immune tone and functional immune states during early tumorigenesis. In addition, systemic factors that disproportionately affect certain populations, including chronic psychosocial stress, metabolic comorbidities, and environmental exposures, may further influence immune function and promote persistent immune dysregulation within premalignant lesions [123,124]. Together, these tumor-intrinsic and extrinsic influences may create conditions that favor earlier or more rapid immunosuppressive microenvironmental remodeling during DCIS progression, thereby increasing susceptibility to invasive transformation as illustrated in Figure 3. These observations highlight the immune microenvironment as a critical intersection between DCIS biology and breast cancer disparities. They underscore the need for integrated, population-informed studies with immune and spatial resolution sufficient to define race-associated immune biomarkers of DCIS progression and to identify opportunities for risk stratification and early intervention.

At the pathway level, immune dysregulation in DCIS progression appears to arise from coordinated alterations in inflammatory signaling, innate immune sensing, and chemokine-mediated cell trafficking. Chronic activation of the NF-κB/IL-6/JAK–STAT axis represents a central mechanism linking inflammation to tumor-promoting immune states, as sustained IL-6 signaling drives STAT3 activation, enhances survival of premalignant epithelial cells, and promotes expansion of immunosuppressive populations such as regulatory T cells and tumor-associated macrophages [33]. In parallel, genomic instability—already detectable in subsets of DCIS—would be expected to activate cytosolic DNA sensing pathways, including the cGAS–STING axis, which normally induces type I interferon responses and supports anti-tumor immunity [129]. However, evidence from invasive breast cancer and other solid tumors indicates that this pathway can become functionally suppressed or rewired during tumor evolution, thereby enabling immune evasion despite persistent DNA damage and chromosomal instability [51]. Complementing these signaling alterations, disruption of chemokine gradients represents a critical mechanism shaping the DCIS immune microenvironment, as altered chemokine expression and transport can impair effective immune cell recruitment, spatial organization, and activation [130]. Together, these intersecting pathways provide a framework linking early genomic instability to immune escape and support a model in which coordinated immune remodeling accompanies and likely contributes to the transition from in situ to invasive disease. While racial disparities in these interconnected pathways remain poorly defined in DCIS, the emerging evidence of race-associated differences in tumor biology and immune contexture makes their systematic investigation not just warranted, but essential to understanding—and ultimately addressing—inequities in DCIS progression and outcomes.

### 6.3. DARC/ACKR1: Racial Disparity in Immune Regulation and DCIS Progression

Emerging evidence also highlights the role of chemokine signaling regulators such as ACKR1 (also known as DARC), which has been linked with immune cell trafficking and tumor-associated inflammation and may contribute to race-associated differences in the breast tumor microenvironment [131]. ACKR1 functions as a non-signaling chemokine receptor that binds and sequesters inflammatory chemokines, helping regulate chemokine gradients, leukocyte trafficking, and angiogenic signaling. In breast cancer, loss or reduced ACKR1 expression has been associated with increased tumor growth, and metastatic potential, largely through elevated availability of pro-inflammatory and pro-angiogenic chemokines such as CXCL1 and CXCL8, which promote recruitment of tumor-associated macrophages and other immunosuppressive cell populations [132,133,134]. By regulating chemokine gradients and leukocyte trafficking, ACKR1 occupies a central position at the interface of immune regulation, inflammation, and angiogenesis, making its loss particularly consequential in shaping a tumor microenvironment to be permissive to invasion.

Importantly, racial differences in chemokine signaling have been reported in breast cancer. Several studies show that chemokine receptors and ligands are differentially expressed across racial groups and molecular subtypes, pointing to broader differences in immune signaling pathways. For example, differential expression of chemokines such as CCL2, CCL5, CXCL9, and CXCL10, along with their associated receptors (e.g., CCR2 and CCR5), has been reported across racial groups, with some of these factors linked to tumor progression and survival disparities [135,136]. Although direct studies in DCIS are limited, these findings suggest that reduced ACKR1 activity could contribute to a more permissive immune microenvironment, potentially facilitating progression from in situ to invasive disease through altered immune cell recruitment and inflammatory signaling.

Beyond its role in chemokine scavenging, ACKR1 also helps shape immune cell trafficking by mediating chemokine translocation and presentation across endothelial barriers, influencing local immune composition and function [137]. This is particularly relevant in the context of breast cancer disparities, as a substantial proportion of individuals of African ancestry carry the Duffy-null allele, which results in loss of erythrocyte ACKR1 expression and altered systemic chemokine buffering [138]. This variation has been associated with higher circulating chemokine levels, chronic inflammatory signaling, and shifts in immune cell dynamics, all of which may influence tumor–immune interactions and contribute to more aggressive disease phenotypes [139,140,141].

Consistent with this, ACKR1-low tumors have been reported more frequently in Black patients and are associated with differences in circulating chemokines and tumor-infiltrating immune cell populations [131]. In parallel, studies have shown that breast tumors in Black women often exhibit higher levels of tumor-infiltrating lymphocytes, particularly CD8+ T cells, although these cells may display features of functional exhaustion, reflecting a more complex immune landscape [142]. Differences in tumor-associated macrophage behavior across ethnic groups have also been observed, further supporting the idea that the immune microenvironment is not uniform across populations [143,144].

Notably, recent integrative reviews have highlighted ACKR1/DARC as a potential mediator of inflammaging, immunosenescence, and breast oncogenesis, particularly in high-risk and racially minoritized populations, further underscoring its relevance as an immune regulatory factor in early breast disease [145]. Collectively, these observations position ACKR1 as a promising yet underexplored mediator of immune regulation in DCIS and a biologically plausible contributor to both progression risk and immune-related racial disparities.

## 7. Kinesin Family Member C1 (KIFC1) and Racial Disparity in DCIS Progression

Increasing attention has turned toward cellular mechanisms that enable early-stage lesions to tolerate genomic instability and thereby promote cancer progression. One such mechanism involves centrosome amplification (CA), in which cells harbor supernumerary centrosomes that disrupt normal bipolar spindle assembly during mitosis. CA can induce aberrant multipolar spindle formation, leading to chromosome missegregation, mitotic catastrophe, and apoptosis. To circumvent this lethal outcome, cancer cells employ centrosome clustering, a process that groups excess centrosomes into two functional spindle poles to permit pseudo-bipolar mitosis [32,146,147].

A key mediator of centrosome clustering is the kinesin motor protein KIFC1 (also known as HSET). KIFC1 crosslinks and slides antiparallel microtubules to generate inward forces that cluster supernumerary centrosomes at opposite sides of the cell, thereby preventing multipolar divisions and enabling successful chromosome segregation [146,148]. Through this mechanism, KIFC1 allows genomically unstable cells to tolerate chromosomal instability while avoiding apoptosis, supporting tumor cell survival, continued proliferation, and the accumulation of additional genomic alterations [148,149]. KIFC1 therefore represents a critical stress-adaptive mechanism that enables survival under conditions of centrosome amplification and chromosomal instability, effectively converting genomic instability from a lethal liability into an evolutionary advantage.

Notably, high KIFC1 expression has been associated with reduced disease-free and overall survival, supporting its role as an active driver of tumor aggressiveness rather than a passive marker of increased proliferation [150]. Importantly, KIFC1 is minimally expressed in most normal tissues but is markedly upregulated in malignant cells with centrosome amplification, highlighting its role as both a marker of aggressive tumor biology and a potential therapeutic vulnerability [147,149].

Although KIFC1 has been most extensively studied in invasive breast cancer, accumulating evidence supports a role for KIFC1 early in breast tumorigenesis, implicating it as a plausible mediator of progression risk in pre-invasive lesions such as DCIS. Centrosome amplification and chromosomal instability have been detected in DCIS—particularly in high-grade and basal-like lesions—indicating that mitotic stress emerges prior to invasion [129,151]. Consistent with this model, a stepwise increase in KIFC1 expression has been reported across the spectrum of breast tumor development, from normal and benign epithelium to atypical ductal hyperplasia (ADH), DCIS, and ultimately invasive carcinoma [152,153]. This progressive upregulation suggests that KIFC1 may be selectively engaged as genomic instability intensifies during tumor evolution. In this context, early induction of KIFC1 could enable genomically unstable DCIS cells to tolerate centrosome amplification and mitotic stress, thereby avoiding mitotic catastrophe and permitting clonal expansion. This hypothesis aligns with clinical observations that DCIS lesions enriched for basal-like features, elevated proliferation, and chromosomal instability exhibit a greater likelihood of progression to invasive breast cancer [120,154].

Accumulating evidence have identified KIFC1 as particularly relevant in aggressive breast cancers that disproportionately affect Black women. Previous studies have proposed centrosome amplification as a mechanistic contributor to racial disparities in breast cancer, citing elevated chromosomal instability, mitotic defects, and aggressive tumor behavior in cancers from Black patients across both invasive disease and DCIS [125]. Notably, DCIS lesions from Black women exhibit higher rates of genomic instability and progression-associated molecular features, suggesting that disparities in mitotic stress and chromosomal instability emerge early in tumorigenesis. Multi-institutional clinical data directly link KIFC1 to racial disparities in TNBC prognosis. Ogden reported significantly higher nuclear localization of KIFC1 in tumors from Black women compared to White women with TNBC, with nuclear KIFC1 serving as an independent biomarker of poor survival in African American patients [126]. These findings are consistent with broader evidence of heightened genomic instability and centrosome amplification in breast cancers arising in Black women. Our group recently demonstrated that KIFC1 expression is significantly elevated in androgen receptor–low and basal-like triple-negative breast cancers (TNBCs), subtypes that are more prevalent among women of African ancestry and associated with poorer clinical outcomes [150]. Together, these studies position KIFC1-mediated centrosome clustering as a key adaptive response to elevated centrosome amplification and chromosomal instability in breast cancers affecting Black women, providing a biologically plausible link between mitotic error tolerance, aggressive disease progression, and potential racial disparity in DCIS progression [125,126].

Importantly, the intersection of KIFC1 biology with racial disparities in breast cancer may reflect contributions from both tumor-intrinsic and systemic influences. Basal-like and hormone receptor-negative DCIS subtypes occur more frequently in Black women, mirroring patterns observed in invasive disease and suggesting that disparities in tumor biology emerge early in carcinogenesis [13,127]. If KIFC1-dependent centrosome clustering represents a conserved survival strategy across disease stages, early engagement of this pathway could bias lesion trajectories toward aggressive phenotypes. In parallel, chronic stress signaling and adaptive stress responses—including metabolic, genomic, and microenvironmental stress—are known to shape survival programs that enable cancer cells to tolerate mitotic disruption and chromosomal instability under adverse conditions [125,128]. Together, these mechanisms position KIFC1 as a compelling candidate biomarker and mechanistic link between early genomic instability and aggressive disease evolution.

However, it is important to note that direct evidence linking KIFC1 expression to progression risk specifically in racially diverse DCIS cohorts remains limited. Much of the existing literature derives from invasive breast cancer or mixed-stage analyses, and although patterns of genomic instability and subtype distribution suggest early pathway engagement, the independent prognostic value of KIFC1 in DCIS has not yet clearly been established.

Accordingly, KIFC1 is best conceptualized as a promising candidate biomarker and mechanistic contributor to progression rather than a definitive driver at the pre-invasive stage. Future studies incorporating racially diverse DCIS cohorts with longitudinal clinical follow-up will be essential to determine whether KIFC1 reliably identifies high-risk lesions and contributes to disparities in disease trajectory.

## 8. Social Determinants of Health and Systemic Drivers of DCIS Progression

### 8.1. Structural Inequities and Cumulative Immune Burden

Structural inequities, embedded within social and economic systems through mechanisms such as residential segregation, discriminatory policy, and unequal access to resources, represent fundamental upstream determinants of health that contribute to persistent racial disparities in cancer outcomes. These forces shape daily living conditions, psychosocial stressors, and environmental exposures that ultimately become biologically embodied, influencing disease risk and progression across the lifespan. Structural racism has been conceptualized as an overarching driver that increases exposure to adverse social determinants of health—including economic disadvantage, lack of neighborhood resources, and chronic stress—which in turn elevate allostatic load and promote immune dysregulation and chronic inflammation, processes known to be implicated in cancer progression and mortality disparities [155,156,157,158]. Several conceptual frameworks illustrate how social determinants “get under the skin.” Biological pathways such as cytokine dysregulation, hypothalamic–pituitary–adrenal (HPA) axis alterations, and innate immune receptor activation link conditions of socioeconomic disadvantage to sustained pro-inflammatory states that drive adverse health outcomes, including cancer [159]. Persistent stress response activation is associated with chronic elevations in inflammatory mediators and impaired cytotoxic immune function, reflecting allostatic overload that undermines protective immunosurveillance [157].

In the context of breast cancer, such biological embedding of structural and psychosocial stressors may establish a systemic environment conducive to tumor-promoting inflammation and immune suppression, thereby influencing early tumor evolution and the likelihood of progression from pre-invasive lesions like DCIS to invasive carcinoma. Epidemiologic evidence further demonstrates that structural measures—such as socioeconomic segregation and systemic disadvantage—are associated with worse cancer incidence and mortality outcomes among racially minoritized populations, supporting the relevance of these upstream determinants to biological and clinical endpoints [160,161].

### 8.2. Chronic Psychosocial Stress and Immune Dysregulation

Chronic psychosocial stress represents a key pathway through which structural inequities are biologically internalized, contributing to breast cancer disparities beyond differences in material resources alone. While structural factors shape exposure, the lived experience of persistent stress—linked to discrimination, racialized vigilance, and socioeconomic instability—drives sustained neuroendocrine responses that engage disease-relevant immune pathways [162,163,164].

Unlike acute stress, chronic psychosocial stress leads to prolonged activation of the HPA axis and sympathetic nervous system, resulting in dysregulated neuroendocrine–immune signaling. Over time, this persistent activation alters immune function in ways that extend beyond elevated inflammatory tone, including impaired glucocorticoid sensitivity, disrupted circadian regulation, and reduced coordination between innate and adaptive immune responses [162,163,164]. Collectively, these changes may compromise immune surveillance while promoting a systemic environment permissive to tumor progression.

Evidence further indicates that stress-related signaling can directly shape the tumor microenvironment. Sympathetic neurotransmitters, particularly norepinephrine, have been shown to enhance angiogenesis, promote epithelial cell survival, remodel stromal and lymphatic compartments, and alter immune cell trafficking in tumor-adjacent tissues [105,106,163,165,166]. In breast cancer models, adrenergic signaling has been linked to increased tumor growth, metastatic potential, and immune evasion, supporting a mechanistic role for stress biology in regulating tumor behavior [105,163,165,166]. Although much of this evidence derives from preclinical systems, convergent findings highlight neural–immune signaling as a conserved pathway linking psychosocial stress to cancer progression.

In early-stage breast lesions, including DCIS, these stress-responsive pathways may reinforce pro-survival signaling and dampen anti-tumor immune activity, contributing to microenvironmental conditions that favor progression. While direct evidence in pre-invasive disease remains limited, this framework identifies a plausible and underexplored connection between chronic psychosocial stress and lesion evolution. Importantly, chronic psychosocial stress intersects with metabolic dysregulation and other exposure-relevant pathways, amplifying downstream biological effects [107,167]. In the United States, Black women experience disproportionately high levels of chronic stress due to structural and interpersonal racism, which may contribute to cumulative immune alterations across the life course [164,167]. Together, these processes provide a biologically coherent framework linking lived social experience to disparities in breast cancer progression and outcomes.

### 8.3. Metabolic Health, Obesity, and Inflammatory Signaling

Diet and metabolic health are increasingly recognized as important modifiers of breast cancer risk and progression, acting through systemic and local mechanisms that influence inflammation, hormone signaling, and cellular metabolism. In DCIS, metabolic dysregulation may shape the biological behavior of early lesions by altering the epithelial and stromal microenvironment before invasion occurs. Obesity, insulin resistance, and chronic low-grade inflammation have all been reported as associated with increased risk of breast cancer recurrence and poorer outcomes, thus suggesting that metabolic state may influence disease trajectory at pre-invasive stages [168,169,170]. Several diet-responsive pathways intersect with processes implicated in DCIS progression. Hyperinsulinemia and elevated insulin-like growth factor signaling promotes epithelial proliferation and survival, while adipose-derived inflammatory cytokines can enhance oxidative stress and DNA damage. In parallel, dietary patterns characterized by high intake of saturated fats and refined carbohydrates have been linked to increased estrogen biosynthesis, altered lipid metabolism, and pro-inflammatory signaling within breast tissue [169,171]. These metabolic perturbations may reinforce aggressive cellular phenotypes, including increased proliferation and resistance to apoptotic cues, thereby creating a permissive environment for DCIS progression through coordinated metabolic and inflammatory signaling.

Racial disparities in metabolic health and nutritional exposure provide important context for understanding differential DCIS outcomes. In the United States, Black women experience higher rates of obesity, metabolic syndrome, and insulin resistance compared with White women, a pattern shaped by structural inequities in food access, neighborhood environments, and chronic stress exposure [172,173]. These disparities reflect long-standing socioeconomic and environmental constraints, which shape both behavioral exposures and biological risk states over the course of life.

Epidemiologic studies have demonstrated that obesity and metabolic dysfunction are more strongly associated with aggressive breast cancer subtypes among Black women, including hormone receptor-negative disease [174,175]. While DCIS-specific data remains limited, these observations suggest that metabolic context may differentially shape the biology of early breast lesions across populations. Importantly, metabolic dysregulation can influence epigenetic programming, inflammatory signaling, and immune surveillance—mechanisms increasingly implicated in DCIS heterogeneity and progression risk [176,177,178]. These findings underscore the need to consider nutrition and metabolic health as biologically relevant contributors to racial disparities in DCIS outcomes, rather than solely as lifestyle correlates.

Systemic metabolic health is a critical determinant of immune function, with obesity and diet-associated inflammation exerting profound effects on immune cell recruitment, activation, and polarization. Obesity is characterized by chronic low-grade inflammation driven by adipocyte hypertrophy, hypoxia, and increased secretion of pro-inflammatory cytokines and adipokines, including IL-6, TNF-α, and leptin [168,169,179]. These factors promote macrophage infiltration and polarization toward immunosuppressive phenotypes while impairing cytotoxic T-cell function and antigen presentation.

At the signaling level, these metabolic and inflammatory cues are mediated through key pathways including STAT3 and NF-κB, which are activated downstream of IL-6 and TNF-α and drive transcriptional programs that promote proliferation, survival, and immune progression in breast cancer [180]. In parallel, insulin resistance and adipocyte-derived signaling converge on the PI3K/AKT/mTOR pathway, enhancing tumor cell growth and metabolic adaption [181]. Additional regulators such as HIF-1α and AMPK further coordinate hypoxic and metabolic stress responses, linking energy imbalance to tumor progression and microenvironmental remodeling [182,183].

In the context of breast cancer, obesity-associated immune dysregulation has been linked to tumor progression and adverse clinical outcomes [168,179]. Experimental studies further demonstrate that adipocytes actively participate in shaping the breast tumor microenvironment by engaging in bidirectional crosstalk with immune cells and epithelial cells, fostering inflammatory and pro-tumorigenic signaling [184,185]. Emerging DCIS-relevant models suggest that similar adipocyte–immune–epithelial interactions may operate during early disease stages, potentially amplifying immune dysfunction and promoting progression in metabolically compromised settings [184]. These findings suggest that disparities in DCIS outcomes may potentially arise from the combined influence of molecular, immune, and environmental factors, which together shape disease progression.

Importantly, the relationship between obesity-associated inflammation and DCIS progression should be interpreted within the broader socioeconomic and environmental context. Obesity, metabolic dysfunction, and dietary patterns are not independent variables but are strongly shaped by structural determinants, including socioeconomic status, food environment, access to healthcare and chronic psychosocial stress [156,157,158,159,160]. These upstream factors influence both metabolic health and inflammatory signaling, introducing important confounding effects when interpreting associations between obesity and cancer outcomes.

As such, while obesity-associated inflammation represents a possible biological contributor to DCIS progression, it may function as a surrogate for cumulative exposure to adverse social and environmental conditions that are promoting chronic inflammation and metabolic dysregulation. In other words, while metabolic inflammation may be a biological mediator of risk, but it is also confounded by structural and lifestyle determinants that must be measured rather than assumed [19,158]. Disentangling these overlapping influences remain a major challenge, and current evidence does not fully resolve the extent to which metabolic differences reflect intrinsic biological effect versus environmentally mediated risk.

### 8.4. Gut Microbiome, Nutrition, and Immune Modulation in DCIS Progression

The gut microbiome has emerged as a critical intermediary linking diet, metabolism, and breast cancer biology. Microbial communities regulate host metabolic function, systemic inflammation, and estrogen metabolism through the enterohepatic circulation. Specific gut bacteria possess β-glucuronidase activity capable of deconjugating estrogens, thereby increasing circulating estrogen levels and potentially influencing hormone-responsive breast tissue [186,187,188]. Diet is a primary determinant of microbiome composition, with high-fat and low-fiber diets associated with reduced microbial diversity and enrichment of pro-inflammatory taxa. Emerging evidence suggests that microbiome dysbiosis may contribute to breast cancer risk and progression by modulating immune tone and metabolic signaling pathways relevant to early tumor development [189]. Although direct studies linking the microbiome to DCIS progression are sparse, alterations in estrogen metabolism, inflammation, and immune regulation provide plausible mechanisms through which microbiome-driven effects could influence pre-invasive lesion behavior.

Notably, racial differences in gut microbiome composition have been reported and are thought to reflect disparities in diet, stress exposure, and environmental factors [190]. Studies have demonstrated that individuals of African ancestry often exhibit higher relative abundance of *Prevotella* species and reduced levels of *Bacteroides*, a pattern associated with high-fiber, plant-rich dietary patterns but also linked to increased inflammatory potential under certain conditions [191,192]. In contrast, populations of European ancestry tend to show *Bacteroides*-dominant microbiomes, which are associated with Western dietary patterns and distinct metabolic outputs [191]. Additional differences in microbial diversity and the abundance of short-chain fatty acid-producing taxa, particularly within the *Firmicutes* phylum, have also been reported, with potential implications for systemic inflammation, estrogen metabolism, and immune regulation [193]. These compositional differences may influence circulating metabolites, including bile acids and microbial-derived estrogens, which are known to impact breast cancer risk and tumor biology [194]. These findings raise the possibility that microbiome-mediated metabolic effects could contribute to population-level differences in DCIS biology and progression risk. However, rigorous, DCIS-specific studies integrating microbiome profiling with molecular characterization of lesions are still lacking.

Together, current evidence supports a model in which diet, metabolic health, and microbiome composition converge to influence the biological landscape of DCIS. While much of the existing literature is derived from invasive breast cancer or population-level risk studies, these pathways are highly relevant to early tumorigenesis and may help explain heterogeneity in DCIS outcomes. Importantly, the intersection of metabolic dysregulation with racial disparities highlights nutrition-related pathways as both biological drivers and modifiable targets. Future studies that integrate dietary assessment, metabolic profiling, microbiome analysis, and molecular characterization of DCIS lesions—particularly in racially diverse cohorts—will be essential for determining how these factors contribute to progression risk. Such approaches may inform prevention strategies and risk stratification frameworks that move beyond tumor-centric features to incorporate systemic and environmental influences on DCIS biology.

### 8.5. Healthcare Access, Screening, and Delays in Intervention

One of the central contributors to racial disparities in breast cancer outcomes is differences in healthcare access, preventative screening, and timely intervention. Disparities in healthcare access; including insurance instability, proximity to care, and availability of specialty services when DCIS is detected and the timeliness of subsequent intervention. Black women in the United States are disproportionately affected by these barriers, contributing to delayed detection, higher rates of symptomatic presentation, and increased risk of progression to invasive disease [101,195].

Screening practices and follow-up adherence are influenced by both structural and psychosocial factors. Historical and ongoing experiences of medical mistrust, systemic bias, and perceived discrimination can reduce engagement with preventive care, even when screening services are technically available [101]. Additionally, competing social and economic demands may impede regular mammographic screening, diagnostic resolution, and timely initiation of treatment. Delays in treatment are particularly consequential for pre-invasive disease. Large population-based analyses demonstrate that Black patients experience higher rates of treatment delays compared with White patients, even after accounting for socioeconomic factors, suggesting the influence of systemic and structural inequities within healthcare delivery [196]. Insurance status and healthcare access are major contributors, with uninsured and underinsured patients facing significantly higher risks of delayed care [197].

## 9. Limitations of Current DCIS Recurrence Risk Prediction Assays

Several commercially available assays are used to estimate recurrence risk and guide adjuvant treatment decisions for DCIS, most notably the Oncotype DX DCIS Score and DCISionRT. While these tools provide clinically relevant prognostic information, they were not designed to resolve immune, inflammatory, or microenvironmental pathways that may be critical to DCIS progression and may contribute to observed racial disparities in outcomes. This limitation is further compounded by fundamental challenges in incorporating microenvironmental features into clinically actionable risk assays.

The tumor microenvironment is inherently dynamic and spatially heterogeneous, with composition varying across regions of the lesion and over time. Unlike tumor-intrinsic signals, microenvironmental features such as immune cell infiltration, stromal interactions, and metabolic gradients are not fully captured by bulk gene expression assays, which average signals across cell populations. Additional challenges include the lack of standardized biomarkers of immune and metabolic states and the limited availability of longitudinal, spatially resolved, and population-representative DCIS datasets. Together, these factors complicate the integration of microenvironmental signals into robust risk stratification tools.

The Oncotype DX DCIS Score (Genomic Health, Inc., Redwood City, CA, USA) primarily captures tumor-intrinsic proliferative and hormone-related biology. Its 12-gene panel includes multiple cell-cycle-associated genes—Ki-67, STK15/AURKA, survivin/BIRC5, CCNB1, and MYBL2—along with PGR, GSTM1, and reference genes, resulting in a score weighted toward cell-cycle activity, progesterone signaling, and stress or detoxification responses [198]. Accordingly, the assay does not directly interrogate biological processes related to immune surveillance or inflammatory signaling, including NF-κB/IL-6/JAK–STAT signaling, cGAS–STING activation, interferon responses, chemokine trafficking, or macrophage and T-cell recruitment, pathways increasingly recognized as contributors to early tumor evolution and progression. Clinical validation studies have demonstrated the assay’s prognostic utility and its ability to stratify benefit from radiotherapy; however, these studies were not designed to capture immune or microenvironment-driven mechanisms of risk [198,199].

DCISionRT (Prelude Dx, Laguna Hills, CA, USA) integrates molecular markers from DCIS lesional tissue with clinicopathologic factors to estimate recurrence risk and to predict benefit from radiotherapy following breast-conserving surgery. The assay incorporates a selected set of proliferation-associated, stromal, and stress-response markers and has demonstrated prognostic and predictive validity across retrospective and prospective cohorts [200,201]. However, despite inclusion of limited inflammatory or microenvironment-adjacent features, DCISionRT similarly lacks pathway-level resolution of innate immune activation, adaptive immune engagement, metabolic reprogramming, and immune–stromal crosstalk—processes that may influence DCIS progression in biologically and population-specific ways.

Collectively, current DCIS recurrence risk assays capture important tumor-intrinsic and selected proliferative or inflammatory features but were not developed to interrogate the biological pathways most likely to underlie race-associated differences in DCIS development and progression. Consequently, existing frameworks may systematically underrepresent, or potentially obscure, mechanisms related to immune signaling, metabolic stress responses, and microenvironmental adaptation that contribute to aggressive disease biology. This limitation likely reflects a broader lack of population-representative, pathway-resolved investigation in early breast cancer and underscores the need for complementary approaches that integrate immune, metabolic, and exposure-informed biology to refine DCIS risk stratification.

Emerging technologies such as multi-omics profiling and digital pathology offer promising approaches to overcome these limitations. Multi-omics platforms integrating genomic, epigenomic, transcriptomic, and proteomic data can resolve pathway-level activity and capture interactions between tumor-intrinsic and microenvironmental processes that are not detectable with single-modality assays. In parallel, digital pathology and spatially resolved transcriptomic approaches enable quantification of immune composition, stromal architecture, and spatial organization within DCIS lesions, providing critical context for understanding progression risk.

In this setting, machine learning and artificial intelligence (AI) approaches are uniquely positioned to reconcile these high-dimensional data types and extract clinically meaningful signals from the tumor immune microenvironment. AI-based models can integrate spatial, molecular, and histopathologic features to identify complex immune–tumor interaction patterns—such as immune exclusion, localized immunosuppression, or inflamed phenotypes—that may not be discernible through conventional analytic methods. By leveraging these multidimensional inputs, machine learning frameworks can uncover latent risk signatures associated with immune contexture and stress-adaptive pathway engagement, enabling more precise stratification of DCIS lesions according to invasive potential.

Importantly, these approaches also create opportunities to incorporate ancestry-informed and population-representative datasets, which may improve the detection of biologically meaningful variation associated with disparities in disease outcomes. Together, these technologies may support the development of next-generation risk models that more accurately capture tumor heterogeneity and microenvironmental dynamics across diverse populations.

## 10. Discussion

The clinical management of DCIS remains a significant challenge with a paucity in reliable biomarkers that can distinguish indolent lesions from those with high invasive potential. Current treatment paradigms are lesion-centered and rely heavily on histopathologic features and receptor status, which do not fully capture the biological heterogeneity of DCIS or account for molecular pathways that may drive progression. The spatial heterogeneity of DCIS poses a persistent problem, and bulk assays may obscure immune/stromal localization effects. Existing cohorts also under-represent, and therefore under-study, DCIS biology in Black women. These limitations can lead to over- or under-treatment, with disproportionate consequences for populations already experiencing poorer outcomes, including Black women in the United States. Black women with DCIS experience higher rates of invasive progression, recurrence, and mortality than White women—differences that are not fully explained by access to care and instead point to underappreciated biological and microenvironmental determinants established at the pre-invasive stage. Identifying biologically informed, equity-conscious biomarkers that refine risk stratification is a critical unmet need.

An important conceptual consideration in interpreting these findings is whether mechanisms such as cellular tolerance of genomic instability represent population-specific biological processes or reflect broader features of aggressive tumor biology. Current evidence supports the latter interpretation. Genomic instability, TP53 dysfunction, and stress-adaptive mechanisms such as centrosome clustering are well-established hallmarks of high-grade breast cancer across populations. However, these features appear to be more frequently enriched in tumors arising in Black women, largely due to differences in molecular subtype distribution, tumor microenvironment contexts, somatic alteration burden, and systemic exposures that shape tumor evolution.

As such, disparities in DCIS progression are unlikely to reflect existence of fundamentally different ancestry-specific biological mechanisms, but rather reflect differential/earlier engagement, stronger selection for, and higher prevalence of shared oncogenic programs. This distinction is critical, as it shifts the focus from categorical racial biology toward an integrated framework in which tumor-intrinsic processes intersect with ancestry, environment, and structural exposures to influence disease trajectory.

Molecular pathways discussed in this article—including Wnt/β-catenin signaling, metabolic reprogramming, immune microenvironment remodeling, and centrosome clustering, represent interconnected processes that may emerge early during breast tumorigenesis. Importantly, these pathways have each been independently associated with aggressive disease features and racial disparities in early-stage and invasive breast cancer, suggesting their potential relevance in DCIS progression. Integrating these biological signals into DCIS risk models could improve prognostication beyond traditional clinicopathologic criteria and enable more personalized treatment strategies. A key translational question is whether stress-adaptive pathways signatures, such as those related to genomic instability tolerance, Wnt signaling, and immune evasion, can be incorporated into clinical decision making for DCIS. In principle, these pathways represent biologically meaningful markers of progression risk, as they capture the capacity of neoplastic cells to survive and evolve under stress conditions. However, several challenges must be addressed before clinical implementation, including the need for robust and reproducible biomarkers, development of assays capable of integrating multi-pathway signals, and validation in longitudinal, population-representative DCIS cohorts. Importantly, current clinical assays are largely optimized for tumor-intrinsic features related to proliferation and hormone-responsiveness, and do not adequately capture these adaptive programs. Nonetheless, incorporation of stress-adaptive signatures into next-generation risk models holds promise for improving risk stratification and enabling more precise, biology-informed therapeutic decision-making, particularly in high-risk populations.

Among these adaptive pathways, KIFC1 represents an actionable candidate for early intervention. As a motor protein essential for centrosome clustering and survival in cells with supernumerary centrosomes, KIFC1 is selectively required in genomically unstable cells and largely dispensable in normal tissue. Importantly, multiple studies, including our recent review, demonstrate a progressive increase in KIFC1 expression from normal and benign breast epithelium through atypical ductal hyperplasia and DCIS, culminating in the highest expression levels in invasive carcinoma. This stepwise elevation suggests that KIFC1 engagement intensifies as genomic instability accumulates during tumor evolution, supporting a functional role in facilitating the transition from pre-invasive to invasive disease. Its association with aggressive breast cancer subtypes, poor prognosis, and enrichment in tumors from Black women, highlights its potential relevance to disparity-associated disease biology. Although direct evidence of KIFC1 function in DCIS remains limited, its mechanistic role in enabling chromosomal instability suggests that KIFC1 expression or activity could mark DCIS lesions with increased invasive potential. Accordingly, future studies evaluating KIFC1 expression in DCIS cohorts with long-term clinical follow-up will be critical for establishing its utility as both a prognostic biomarker and a potential therapeutic target in the pre-invasive setting.

Beyond tumor-intrinsic mechanisms, systemic factors such as nutrition and gut microbial composition may further influence genomic instability and stress-adaptive pathways relevant to KIFC1 biology. Diet-induced metabolic stress, chronic inflammation, and microbiome-derived metabolites have been shown to modulate oxidative stress, DNA damage responses, and chromatin organization—processes that directly impact centrosome integrity and mitotic fidelity. Notably, disparities in dietary patterns, food access, and gut microbiota composition have been documented across racial and socioeconomic groups and have been linked to differences in breast cancer risk and tumor aggressiveness. These systemic influences may exacerbate mitotic and metabolic stress in pre-invasive lesions, increasing reliance on stress-mitigation mechanisms such as KIFC1-mediated centrosome clustering. Integrating nutritional and microbiome factors into studies of DCIS progression may therefore provide additional insight into how extrinsic pressures intersect with tumor-intrinsic adaptations to drive aggressive disease evolution. Thus, stress-adaptive signatures such as KIFC1/centrosome amplification, TP53/CNA burden, immune-exclusion markers, and metabolic-inflammatory signatures could even-tually complement existing assays, but they require prospective validation, assay stand-ardization, calibration across diverse populations, and evidence that they improve deci-sion-making beyond grade, ER status, margins, age, Oncotype DX DCIS Score, and DCISionRT. Multi-omics and digital pathology can further integrate tumor genomics, methylation, transcriptomics, multiplex immune phenotyping, spatial localization, and histologic architecture in the same lesion. Digital pathology can potentially quantify stro-mal organization, immune infiltration, myoepithelial integrity, and adipose inflammation at scale. Race-conscious implementation should require calibration by genetic ancestry, self-identified race, treatment exposure, tissue composition, and social determinants rather than assuming that a single biomarker threshold performs equally across populations.

In this context, machine learning and artificial intelligence (AI)-based approaches provide a powerful framework for integrating these multidimensional data streams into clinically actionable models. AI-driven methods can synthesize stress-adaptive pathway signatures—such as genomic instability tolerance, immune exclusion, metabolic reprogramming, and epigenetic remodeling—with spatial and clinicopathologic features to identify latent patterns of progression risk not captured by conventional approaches. Importantly, these models can incorporate spatially resolved digital pathology data and multi-omic profiles at scale, enabling more refined classification of DCIS lesions along a continuum of invasive potential. When rigorously developed and validated in diverse, population-representative cohorts, such approaches have the potential to improve risk stratification, support personalized treatment decisions, and reduce overtreatment. However, consistent with the principles outlined above, careful attention to training data composition, bias mitigation, and equitable model calibration will be essential to ensure that AI-based tools enhance—rather than exacerbate—existing disparities in breast cancer outcomes.

Despite the integrative framework presented in this article, several limitations should be acknowledged. First, much of the mechanistic evidence discussed is derived from studies of invasive breast cancer or model systems, with relatively limited direct validation in DCIS, particularly in racially diverse cohorts. Second, reliance on self-identified race as a variable introduces inherent limitations, as it does not fully capture genetic ancestry, environmental exposures, or social determinants that shape disease biology. Third, the review synthesizes findings across heterogeneous study designs, platforms, and populations, which may introduce variability in interpretation. Finally, key areas such as tumor microenvironment dynamics, stress-adaptive signaling, and metabolic influences in DCIS remain incompletely characterized, highlighting the need for longitudinal, multi-omic, and population-representative studies to validate and extend these concepts.

## 11. Conclusions

Ductal carcinoma in situ encompasses a biologically heterogeneous group of lesions, only a subset of which progress to invasive breast cancer. Despite advances in screening and pathology, current risk stratification strategies remain limited in their ability to distinguish indolent disease from lesions with true malignant potential, contributing to substantial overtreatment. The evidence reviewed here supports a model in which early genomic instability, immune dysregulation, and cellular stress-tolerance mechanisms interact to shape the evolutionary trajectories of high-risk lesions.

A recurring theme across these pathways is not simply the presence of genomic instability, but the ability of early neoplastic cells to tolerate and adapt to it. Mechanisms such as centrosome amplification-associated clustering mediated by KIFC1 enable survival under otherwise lethal mitotic stress, facilitating clonal persistence and the accumulation of additional oncogenic alterations. In parallel, immune-modulatory pathways—including chemokine buffering mediated by ACKR1/DARC—may influence local inflammatory tone and immune surveillance, thereby shaping the selective pressures acting on emerging lesions. Together, these findings suggest that progression risk may be governed less by isolated molecular alterations than by coordinated programs that permit continued survival in genomically and immunologically stressful environments.

Importantly, these adaptive pathways may not be uniformly engaged across patient populations. Racial disparities in breast cancer outcomes, including higher rates of aggressive subtypes and worse clinical prognosis among racially minoritized populations, raise the possibility that tolerance-based mechanisms of genomic instability may be differentially relied upon in early disease. In this context, race should not be viewed as a biological determinant, but rather as a proxy for cumulative exposures—such as chronic inflammation, immune dysregulation, and structural stressors—that can influence tumor evolution. Failure to incorporate this dimension risks obscuring biologically meaningful heterogeneity within DCIS and may contribute to inequities in risk assessment and clinical management.

Collectively, these observations argue for a shift toward an integrated, disparities-informed framework for studying early breast cancer progression. Future efforts combining spatially resolved genomics, immune profiling, and longitudinal sampling across diverse populations will be essential to determine whether stress-tolerance adaptive pathways reliably predict progression risk and can be leveraged to guide risk-adapted care. A deeper understanding of how early lesions survive genomic stress—rather than succumb to it—may ultimately enable more precise, equitable management strategies that reduce overtreatment while preserving oncologic safety. Advancing such approaches is essential not only for improving precision in DCIS management, but for addressing entrenched disparities in breast cancer outcomes at their earliest stage.

## 12. Future Directions

From a translational perspective, these findings underscore the need for prospective, well-annotated DCIS cohorts that include diverse patient populations and integrate molecular, metabolic, and immune profiling. Equity-focused study design is particularly important, as many existing datasets underrepresent Black women and other historically marginalized groups, limiting the ability to disentangle biological contributors to disparity from social and structural determinants of health. Incorporating ancestry-informed analyses, while avoiding biological essentialism, may help clarify how molecular pathway activation intersects with environmental exposures and systemic inequities to shape disease trajectory.

Finally, the identification of early, targetable vulnerabilities in DCIS raises the possibility of intervention prior to progression. Rather than uniformly escalating or de-escalating treatment, biologically informed risk stratification could enable tailored approaches, including surveillance for truly indolent lesions and targeted therapies for high-risk disease. By focusing on pathways that link genomic instability, metabolism, immune evasion, and racial disparities, future research has the potential not only to improve DCIS outcomes but also to reduce inequities in breast cancer morbidity and mortality. DCIS represents a critical window for intervention and advancing our understanding of its molecular heterogeneity is essential for achieving more precise and equitable breast cancer care.

## Figures and Tables

**Table 1:** Molecular biomarkers and signaling pathways with reported racial differences in DCIS and early breast cancer.

Biomarker/Pathway	Biological Role	Mechanism in DCIS Progression	Reported Racial Differences	Clinical Relevance	Therapeutic Strategies
TP53	Tumor suppressor	Loss-of-function mutations promote genomic instability and progression from in situ to invasive disease	In breast tumors from Black patients, TP53 mutations and dysfunctional p53 signaling are more frequently observed, consistent with enrichment of high-grade, basal-like phenotypes and more aggressive biology	Strongly associated with high-grade DCIS, early recurrence, and poor prognosis	Restoration of p53 signaling, synthetic lethal approaches, and targeting downstream DNA damage response vulnerabilities
Copy-number alteration burden/chromosomal instability	Genomic architecture and clonal diversity	Increased CNA burden facilitates tumor evolution, heterogeneity, and invasive potential	Higher levels of genomic instability and recurrent CNAs have been reported in tumors from Black women, including gains in MYC and ERBB2 and losses in TP53 and RB1	Predictive of aggressive disease behavior and progression risk	Risk stratification; monitoring clonal evolution and progression
Ki-67	Proliferation marker	High expression reflects rapid tumor cell cycling and progression risk	Elevated Ki-67 indices are more commonly reported in DCIS and invasive tumors from Black patients, consistent with increased proliferative drive and aggressive disease	Predictor of recurrence, progression, and treatment response	Risk-adapted escalation of therapy and intensified surveillance in highly proliferative lesion
Estrogen receptor signaling (ESR1, PGR, BCL2)	Hormone-dependent growth regulation	Loss or attenuation of ER signaling reduces endocrine responsiveness	Racial differences in hormone receptor expression and downstream signaling reported, though variable in DCIS cohorts	Influences subtype classification and therapy response	Guides endocrine therapy eligibility and prevention strategies
HER2/ERBB2	Receptor tyrosine kinase	Amplification promotes sustained proliferative and survival signaling	Racial variation in HER2 amplification frequency and subtype distribution reported	Predictive biomarker for recurrence risk and targeted therapy	HER2-directed agents such as monoclonal antibodies, ADCs, and TKIs where applicable
CD8+ T cells	Cytotoxic lymphocytes mediating anti-tumor immunity	Limit tumor progression through immune-mediated cell killing	Tumors from Black patients often demonstrate reduced effective CD8+ T-cell function or increased immune exhaustion despite variable infiltration levels	Associated with immune surveillance and response to immunotherapy	Immune checkpoint inhibitors and strategies to enhance T-cell activation and persistence such as adoptive t cell therapy
Tumor-associated macrophages (M2-like)	Immunosuppressive myeloid cells	Promote angiogenesis, matrix remodeling, and immune suppression	Increased M2 macrophage polarization and density have been observed in tumors from Black patients, contributing to a more immunosuppressive microenvironment	Linked to progression, immune evasion, and poorer outcomes	CSF1R inhibitors; macrophage reprogramming
ACKR1 (DARC)	Chemokine scavenging and immune regulation	Regulates chemokine gradients, leukocyte trafficking, and inflammation	Reduced expression and ancestry-linked genetic variants more common in individuals of African ancestry	Modifier of tumor–immune interactions and progression risk	Immune-modulating strategies; risk stratification
CXCL1, CXCL8 (IL-8)	Pro-inflammatory chemokines	Recruit myeloid cells and promote tumor-associated inflammation	Differential expression reported across racial groups	Associated with immune suppression and invasiveness	Targeting chemokine signaling axes
CCL2, CCL5, CXCL9, CXCL10	Immune cell recruitment	Shape tumor immune composition and inflammatory tone	Differential expression associated with outcome disparities	Linked to immune contexture and prognosis	Immune and inflammatory pathway modulation

**Table 2:** Proposed biomarkers and pathways contributing to racial disparity DCIS invasiveness and progression. Hypothesis-driven integration based on DCIS-specific data, invasive breast cancer literature, and mechanistic plausibility.

Biomarker/Pathway	Biological Role	Mechanism in DCIS Progression	Rationale for Contribution to Racial Disparities	Clinical Relevance	Therapeutic/Translational Implications
KIFC1 (HSET)-mediated centrosome amplification tolerance	Mitotic stress-adaptive pathway enabling centrosome clustering and survival in the presence of supernumerary centrosomes	KIFC1 clusters supernumerary centrosomes to prevent multipolar mitosis, enabling survival and proliferation of genomically unstable cells	Elevated KIFC1 expression reported in aggressive tumors from Black women; higher genomic instability burden may increase reliance on centrosome tolerance mechanisms (DCIS-specific racial data limited)	Marker of chromosomal instability tolerance and aggressive disease evolution	Small-molecule KIFC1 inhibitors (e.g., SR31527, CW069, AZ82, PJ34); targeting mitotic stress tolerance to prevent invasive progression
Canonical Wnt/β-catenin signaling	Cell fate determination and stemness	Promotes proliferation, EMT-like programs, and invasion	Enhanced activation reported in TNBC affecting Black women, direct DCIS data lacking	Linked to invasiveness and immune exclusion	Wnt pathway inhibitors such as IWP-4, XAV-939, and iCTR; differentiation therapies
Epigenetic silencing of Wnt antagonists (SFRP1, SFRP2, SFRP5, DKK1)	Negative regulation of Wnt signaling	Loss of antagonism permits sustained pathway activation	Epigenetic regulation influenced by environmental and inflammatory exposures	Predicts poor prognosis and aggressive behavior	Epigenetic therapies; biomarker development
Wnt-mediated immune exclusion	Immune–oncogenic crosstalk	Suppresses dendritic cell recruitment and cytotoxic T-cell infiltration	Racial differences in Wnt signaling may indirectly shape immune disparities	Links oncogenic signaling to immune evasion	Combination oncogenic–immune targeting
NF-κB signaling	Inflammatory transcriptional regulation	Sustains pro-tumor inflammation and survival signaling	Transcriptomic enrichment of inflammatory pathways in tumors from Black women	Associated with invasiveness and therapy resistance	Anti-inflammatory and pathway-targeted strategies
JAK/STAT (IL-6-driven)	Cytokine signaling and immune modulation	Promotes immunosuppressive and proliferative programs	Elevated inflammatory signaling linked to chronic stress and metabolic burden	Potential driver of immune dysfunction in DCIS	Cytokine and pathway inhibition such as tocilizumab and tofacitinib
DNA methylation field effects	Epigenetic regulation	Alters gene expression prior to invasion	Race-associated methylation differences observed in tumors and normal-adjacent tissue	Early biomarker of progression risk	Epigenetic risk stratification
Metabolic inflammation (IL-6, TNF-α, leptin)	Systemic immune modulation	Enhances oxidative stress, proliferation, and immune suppression	Higher prevalence of obesity and metabolic dysfunction in Black women	Links systemic physiology to lesion biology	Lifestyle, metabolic, and anti-inflammatory interventions
Gut microbiome dysbiosis	Immune and metabolic regulation	Influences estrogen metabolism and inflammatory tone	Racial differences in microbiome composition reported	Indirect modifier of progression risk	Dietary and microbiome-targeted strategies

## Data Availability

No new data were created or analyzed in this study. Data sharing is not applicable to this article.
